# Lymphadenopathy after *BNT162b2* Covid-19 Vaccine: Preliminary Ultrasound Findings

**DOI:** 10.3390/biology10030214

**Published:** 2021-03-11

**Authors:** Vincenza Granata, Roberta Fusco, Sergio Venanzio Setola, Roberta Galdiero, Carmine Picone, Francesco Izzo, Roberta D’Aniello, Vittorio Miele, Roberta Grassi, Roberto Grassi, Antonella Petrillo

**Affiliations:** 1 Radiology Division, “Istituto Nazionale Tumori IRCCS Fondazione Pascale—IRCCS di Napoli”, 80131 Naples, Italy; v.granata@istitutotumori.na.it (V.G.); s.setola@istitutotumori.na.it (S.V.S.); r.galdiero@istitutotumori.na.it (R.G.); c.picone@istitutotumori.na.it (C.P.); a.petrillo@istitutotumori.na.it (A.P.); 2 Hepatobiliary Surgical Oncology Division, “Istituto Nazionale Tumori IRCCS Fondazione Pascale—IRCCS di Napoli”, 80131 Naples, Italy; f.izzo@istitutotumori.na.it; 3 Hospital Pharmacy Division, “Istituto Nazionale Tumori IRCCS Fondazione Pascale—IRCCS di Napoli”, 80131 Naples, Italy; r.daniello@istitutotumori.na.it; 4 Division of Radiodiagnostic, “Azienda Ospedaliero-Universitaria Careggi”, 50139 Firenze, Italy; vmiele@sirm.org; 5 Division of Radiology, University of Campania Luigi Vanvitelli, 80125 Naples, Italy; robertagrassi89@gmail.com (R.G.); roberto.grassi@unicampania.it (R.G.); 6 Foundation SIRM, 20122 Milan, Italy

**Keywords:** COVID-19 infection, vaccine, ultrasound, lymphadenopathy

## Abstract

**Simple Summary:**

Eccentric cortical thickening with wide echogenic hilum and oval shape, asymmetric eccentric cortical thickening with wide echogenic hilum and oval shape, concentric cortical thickening with reduction in the width of the echogenic hilum and oval shape, and huge reduction and displacement of the echogenic hilum and round shape are the features that we found in post *BNT162b2* Covid-19 Vaccine lymphadenopathies.

**Abstract:**

During a spontaneous and autonomous study, we assessed the ultrasound finding of lymphadenopathy after *BNT162b2* Pfizer vaccine. We enrolled 18 patients with 58 lymphadenopathies: in 10 patients, they were in the laterocervical side, while in 8 patients in the axillar site. The largest diameter was 16 mm with a range from 7 to 16 mm (median value = 10 mm). In the same patient, we found different ultrasound nodal findings. A total of 25 nodes showed eccentric cortical thickening with wide echogenic hilum and oval shape. In total, 19 nodes showed asymmetric eccentric cortical thickening with wide echogenic hilum and oval shape. Overall, 10 nodes showed concentric cortical thickening with reduction in the width of the echogenic hilum and oval shape. A total of four nodes showed huge reduction and displacement of the echogenic hilum and round or oval shape. No anomaly was found at the Doppler echocolor study. In conclusion, eccentric cortical thickening with wide echogenic hilum and oval shape, asymmetric eccentric cortical thickening with wide echogenic hilum and oval shape, concentric cortical thickening with reduction in the width of the echogenic hilum and oval shape, and a huge reduction and displacement of the echogenic hilum and round shape are the features that we found in post *BNT162b2* Covid-19 Vaccine lymphadenopathies.

## 1. Background

In December 2019, health authorities in Wuhan, China, identified a cluster of acute respiratory disease of unknown etiology [1]. Subsequently the researchers identified a new viral agent, SARS-CoV-2, as responsible for the heart of an international outbreak centered in Hubei. On 30 January 2020, World Health Organization (WHO) confirmed the COVID-19 epidemic as a public health emergency and on 11 March 2020 demarcated the rapid spread of infection as a pandemic in the world [1,2]. Globally, at the time of writing (17 January 2021), there have been 93,194,922 confirmed cases of COVID-19, including 2,014,729 deaths, reported to WHO. In Italy, there have been 2,368,733 confirmed cases of COVID-19 with 81,800 deaths. Up to today, effective treatment has not yet been developed so that mechanical respiratory support is the only therapy in critically ill patients [3,4,5]. In this scenario, it was necessary to develop a vaccine as soon as possible, to prevent coronavirus disease 2019 and to protect persons who are at high risk for complications. At the time of writing in Italy have been approved two vaccines: the mRNA-1273 vaccine Moderna [6] and BNT162b2 Pfizer drug [7]. In 27 December, health authorities in Italy have authorized the administration of the *BNT162b2* Pfizer vaccine in healthcare personnel, defining this day as Vaccine (V)-day. 

During the trial clinic on *BNT162b2*, vaccine data on local and systemic reactions and use of medication were collected with electronic diaries from participants in the reactogenicity subset (8183 participants) for 7 days after each vaccination [7]. Adverse event analyses are provided for all enrolled participants, with variable follow-up time after dose. More *BNT162b2* recipients than placebo recipients reported any adverse event (27 and 12%, respectively) or a related adverse event (21 and 5%). A total of 64 vaccine recipients (0.3%) and 6 placebo recipients (<0.1%) reported lymphadenopathy. We assessed ultrasound (US) findings in 18 consecutive patients from healthcare personnel that reported lymphadenopathy after *BNT162b2* Pfizer drug.

## 2. Materials and Methods

### 2.1. Patient Population

This is a spontaneous and autonomous study, without the authorization of the ethics committee, for which patients have allowed data processing in accordance with National Privacy Regulations [8]. This observational study included the period from 27 December 2020 to 16 January 2021, the date of the last administration of the vaccine first dose among the health personnel in Campania. In this time range, 18 consecutive patients from healthcare personnel who received the first dose of *BNT162b2* Pfizer vaccine were enrolled: 10 for palpable mass appeared after the vaccine, 8 sent to the ultrasound study by the pharmacovigilance physician.

In Table 1 we reported the patients study group characteristics.

### 2.2. US Protocol and Images Analysis

Nodes ultrasound exams were performed by dedicated radiologists, using RS85 Samsung System (Samsung Healthcare GmbH, Schwalbach, Germany) in combination with a linear 5 to 12-MHz array transducer. 

A total of four in-site expert radiologists in interpretation of nodal images, recorded the data in consensus. Presence, side, size, shape, echogenicity, cortex feature, margin, and hilum of the lesions were categorized. We also assessed color doppler features.

## 3. Results

We assessed 18 patients with 58 lymphadenopathy after *BNT162b2* Covid-19 Vaccine.

In 10 patients (55.5%) they were in laterocervical side while 8 (44.5%) in the axillar site. The largest diameter was 16 mm with a range from 7 to 16 mm (median value = 10 mm). In the same patient we found different ultrasound nodal findings.

No anomaly was found on the Doppler echo-color study.

**Figure 1:**
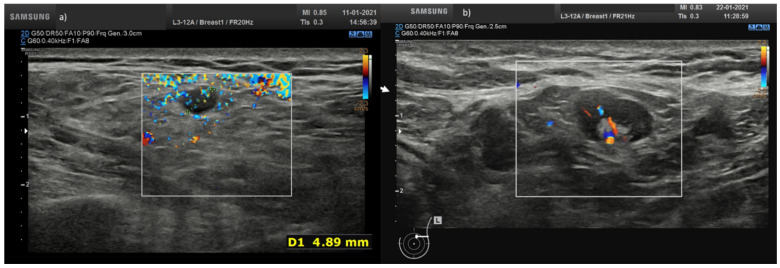
(**a**) Hypoechoic lymph node round shape without hilum (arrow) in laterocervical side; (**b**) axillary lymph node with concentric cortical thickening with reduction in the width of the echogenic hilum and oval shape.

**Figure 2:**
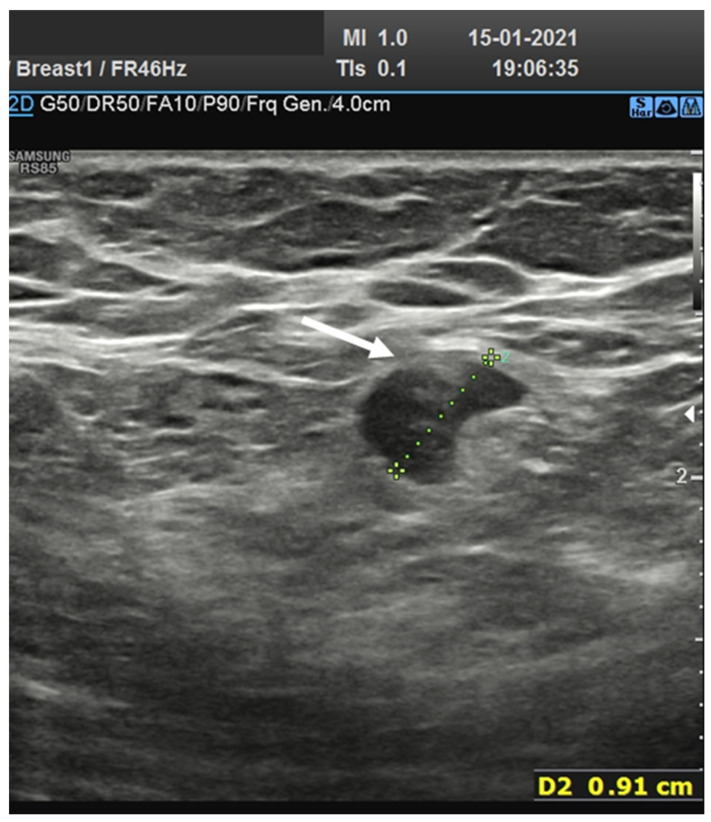
Axillary lymph node with eccentric asymmetric cortical thickening with wide echogenic hilum and oval shape.

A total of 25 (43.1%) nodes showed eccentric cortical thickening with wide echogenic hilum and oval shape (Figure 1 and Figure 2). 

**Figure 3:**
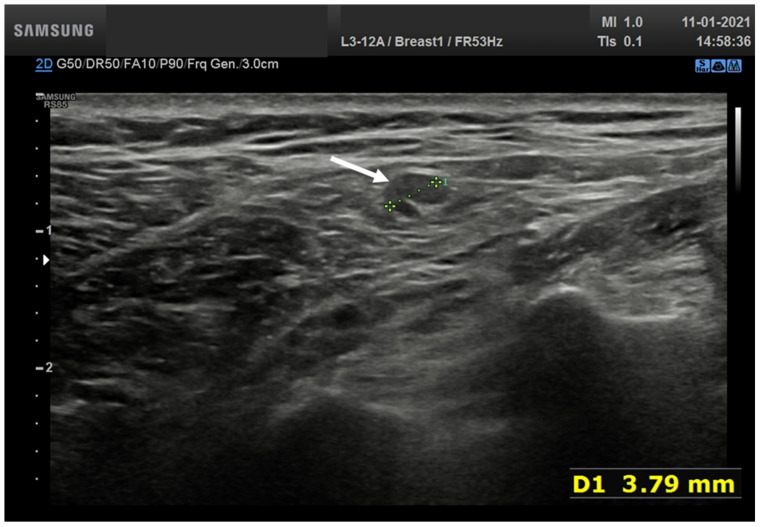
Lymph node (arrow) with asymmetric eccentric concentric cortical thickening in laterocervical side with reduction in the width of the echogenic hilum and oval shape.

Overall, 19 nodes (32.8%) showed asymmetric eccentric cortical thickening with wide echogenic hilum and oval shape (Figure 3).

A total of 10 nodes (17.2%) showed concentric cortical thickening with reduction in the width of the echogenic hilum and oval shape (Figure 1b).

**Figure 4:**
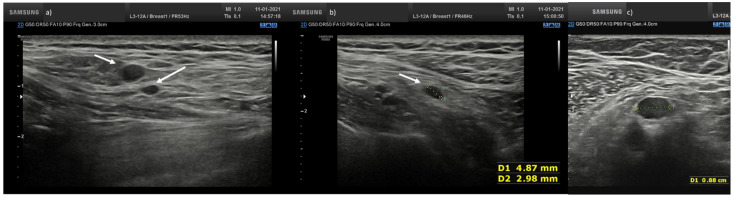
(**a**) Hypoechoic lymph node round shape without hilum (arrows) in laterocervical side; (**b**) Hypoechoic lymph node oval shape without hilum (arrow) in laterocervical side; (**c**) Axillary small hypoechoic lymph node oval shape without hilum (arrow).

In total, four nodes (6.9%) showed huge reduction and displacement of the echogenic hilum and round or oval shape (Figure 4).

## 4. Discussion

Recent results have revealed the efficiency of some imaging methods, including chest radiographs and chest computed tomography scans, in the management of COVID-19 disease [9,10,11,12,13,14,15,16,17,18,19,20,21,22,23,24,25,26,27,28,29,30]. Instead, at the best of our knowledge, this is the first paper describing the appearance of nodes after *BNT162b2* Covid-19 vaccine. Although the onset of lymphadenopathy after vaccine is known, Polack et al reported a frequency of only 0.3% [29]. However, we think that the date may be underestimated, in consideration of our sample size, although it is a representative population of a small community. Moreover, although in 10 patients the lymph nodes were painful, in four patients the data were an occasional finding diagnosed during a US examination performed for another reason. 

US is commonly regarded as the imaging modality of choice in the assessment of palpable soft-tissue abnormalities. According to the appropriateness criteria of the American College of Radiology, US is “usually appropriate” to evaluate superficial or palpable soft-tissue masses, while magnetic resonance imaging (MRI) is “usually appropriate” in case of non-diagnostic initial US evaluation [31], while CT is appropriate for assessment of systemic diseases [32].

Lymph node cortical thickness and uniformity are the most important criteria for distinguishing between normal and abnormal nodes. Normal lymph nodes have a reniform shape, a uniformly hypoechoic cortex with a maximal thickness of 3 mm, smooth margins, and a central fatty hilum [33]. Findings of cortical thickness in excess of 3 mm, eccentric thickening, irregular margins, and encroachment on or displacement of the fatty hilum are suggestive of a pathologic process [33]. 

We found different US findings in the same patient; 43.1% of lymph nodes showed eccentric cortical thickening with wide echogenic hilum and oval, 32.8% of lymph nodes showed asymmetric eccentric cortical thickening with wide echogenic hilum and oval shape. A total of 17.2% of lymph nodes showed concentric cortical thickening with reduction in the width of the echogenic hilum and oval shape and 6.9% showed huge reduction and displacement of the echogenic hilum and round or oval shape.

We believe it is important to know and recognize these structural alterations, since as soon as the vaccine will be also available to non-healthcare personnel, it is important for a radiologist to be able to identify post-vaccine lymphadenopathy compared to lymphadenopathy for another cause, especially in cancer patients.

There are multiple limitations of this work. First of all, the small and spontaneous sample, consequently we did not know the real incidence of lymphadenopathy. Second, not having performed an elastosonographic and echocontrastographic examination; finally, the absence of a pathological correlation.

Future objective is an evaluation of the entire population of our institution undergoing vaccination for a correct estimate of the incidence and a more complex ultrasound analysis.

## 5. Conclusions

Eccentric cortical thickening with wide echogenic hilum and oval shape, asymmetric eccentric cortical thickening with wide echogenic hilum and oval shape, concentric cortical thickening with reduction in the width of the echogenic hilum and oval shape, and huge reduction and displacement of the echogenic hilum and round shape are the features that we found in post *BNT162b2* Covid-19 Vaccine lymphadenopathies.

## Figures and Tables

**Table 1:** Patients study group characteristics.

Patient (Age; Sex)	Vaccine Administation Date	Time of Appearance/Disappearance	Number of Nodes	Side	Medication Use	Others Symptoms	Previous Covid Infection (Time)	Presence of Antibodies
1 (47 y; M)	01/04	36 h/5 days	3	laterocervical side	Yes	No	No	No
2 (63 y; F)	01/08	24 h/3 days	5	laterocervical side	Yes	Fever (37.5 °C); headache; fatigue; diarrhea	No	No
3 (38 y; F)	01/07	24 h/3 days	3	laterocervical side	No	No	No	No
4 (43 y; F)	01/06	12 h/24 h	4	laterocervical side	No	No	No	No
5 (42 y;F)	01/15	24 h/4 days	3	Axilla	No	No	No	No
6 (35 y; F)	01/15	12 h/24 h	3	laterocervical side	No	Fever (38 °C)	Yes	Yes
7 (54 y; M)	01/02	36 h/73 days	1	Axilla	No	Fever (37.5 °C)	Yes	No
8 (49 y; F)	06/01	36 h/5 days	3	Axilla	No	No	No	No
9 (42 y; F)	01/02	24 h/4 days	4	Axilla	Yes	Fever (37.5 °C)	No	No
10 (41 y; F)	01/05	36 h/5 days	1	laterocervical side	No	Fever (37.5 °C)	No	No
11 (56 y; M)	01/15	36 h/5 days	3	laterocervical side	No	Fever (38 °C)	Yes	Yes
12 (47 y; M)	01/04	24 h/ 3 days	4	Axilla	No	No	No	No
13 (63 y; F)	01/15	24 h/3 days	5	laterocervical side	Yes	Fever (37.5 °C); headache; fatigue; diarrhea	No	No
14 (35 y; F)	01/13	24 h/3 days	3	Axilla	No	No	No	No
15 (40 y; F)	01/05	24 h/3 days	4	Axilla	No	Fatigue	No	No
16 (61 y; F)	01/10	24 h/3 days	5	laterocervical side	Yes	Headache; fatigue; diarrhea	No	No
17 (51 y; M)	01/08	36 h/5 days	3	Axilla	No	No	No	No
18 (26 y; F)	01/10	24 h/ 3days)	1	laterocervical side	No	No	No	No
